# Supplementary material for: PGC-1α4 Interacts with REST to Upregulate Neuronal Genes and Augment Energy Consumption in Developing Cardiomyocytes
Source: Cells. 2022 Sep 20;11(19):2944. doi: 10.3390/cells11192944 (PMC9564192; doi:10.3390/cells11192944)
Supplement: Supplementary file 1 [file cells-11-02944-s001.zip › cells-1905913-supplementary.pdf]

Supplementary material

PGC-1α4 interacts with REST to upregulate neuronal genes and augment energy consumption in developing cardiomyocytes

Tomi Tuomainen [1], Nikolay Naumenko [1], Maija Mutikainen [1], Anastasia Shakirzyanova [1], Sarah Sczelecki [2], Jennifer L. Estall [2], Jorge L. Ruas [3], Pasi Tavi [1,*]

[1] A.I. Virtanen Institute for Molecular Sciences, University of Eastern Finland, 70210 Kuopio, Finland
[2] Institut de Recherches Cliniques de Montréal, Montréal, QC H2W 1R7, Canada
[3] Molecular and Cellular Exercise Physiology, Department of Physiology and Pharmacology, Biomedicum, Karolinska Institutet, 17165 Stockholm, Sweden
* Correspondence: pasi.tavi@uef.fi

Supplementary figures

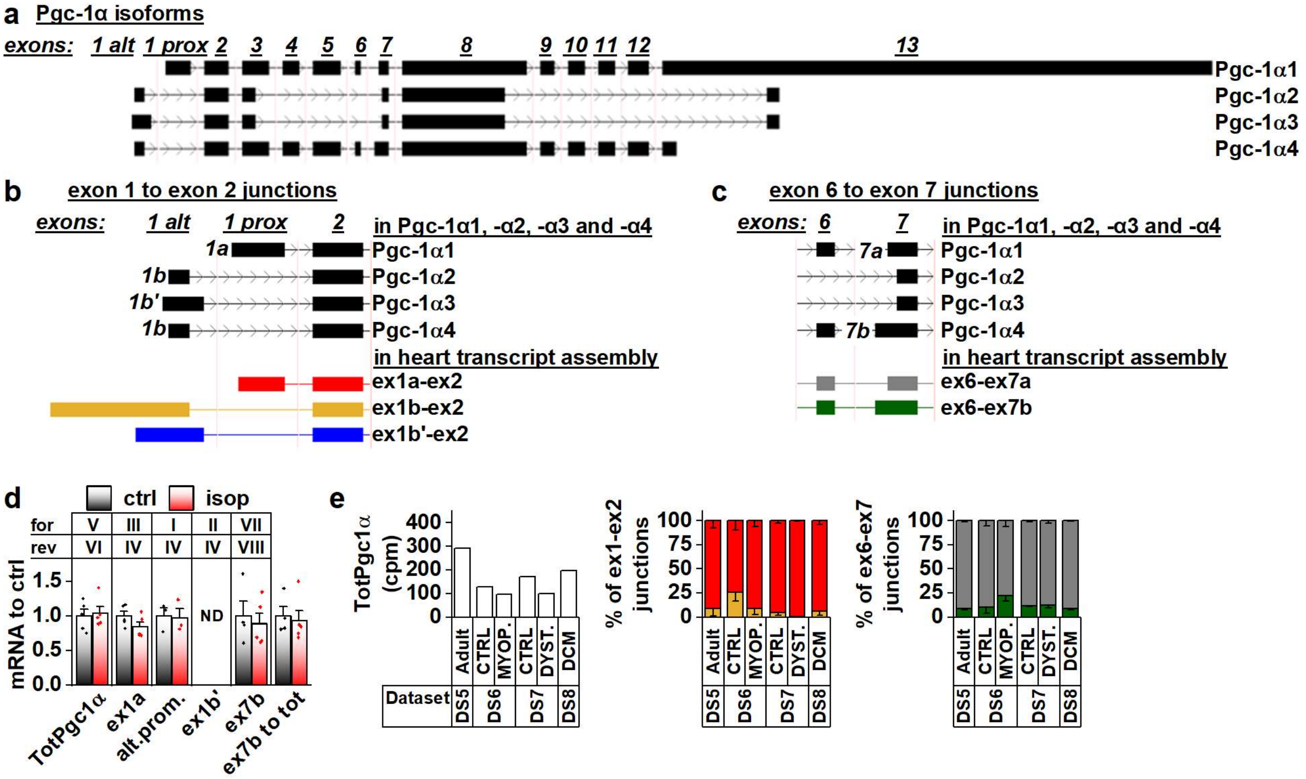

Figure S1. Splicing and promoter usage of *Pgc-1α* gene in the mouse heart. (a) Exon structures of previously reported mouse *Pgc-1α* isoforms. Sequences for *Pgc-1α1*, *Pgc-1α2*, *Pgc-1α3* and *Pgc-1α4* were acquired from the GenBank database (accession id's NM_008904, JX866946, JX866947 and JX866948, respectively). **(b)** Enlarged view of *Pgc-1α* exon structures showing the junctions between exons 1 and 2. Structures colored red, yellow and blue show exon 1 to exon 2 junctions that were present in transcript assemblies from mouse heart RNA sequencing datasets. **(c)** Enlarged view of *Pgc-1α* exon structures showing the junctions between exons 6 and 7. Structures colored grey and green show exon 6 to exon 7 junctions that were present in transcript assemblies from mouse heart RNA sequencing datasets. **(d)** *Pgc-1α* expression analysis with qRT-PCR from isoprenaline-exposed E12.5 mouse cardiomyocytes (n=6). See Figure 1A for PCR primers (for/rev) used to detect expression of specific exons. **(e)** Total *Pgc-1α* expression (*top*), ratios of reads aligning to exon 1-exon 2 junctions (*middle*) and ratios of reads aligning to exon 6-exon 7 junctions (*bottom*) assessed from publicly available RNA sequencing datasets from human hearts.

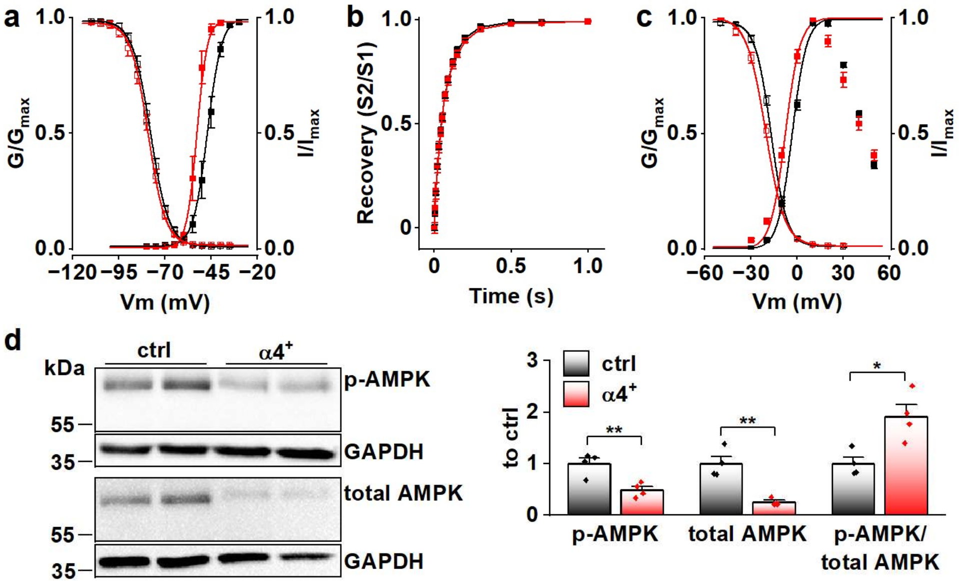

Figure S2. Sodium and L-type calcium current kinetics in 4-week-old α4+ cardiomyocytes. (a) Voltage dependent activation (closed squares) and inactivation (open squares) of sodium current (Boltzmann fitting, see the fitting result in Supplementary Table S2) (n[ctrl]=13, n[$\alpha 4^+$]=12). **(b)** Time dependent recovery of sodium current (n[ctrl]=13, n[$\alpha 4^+$]=12). **(c)** Voltage-dependent activation (closed squares) and inactivation (open squares) of L-type calcium current (Boltzmann fitting, see the fitting result in Supplementary Table S2) (n[ctrl]=30, n[$\alpha 4^+$]=36). **(d)** Representative western blots showing expression of phosphorylated AMP kinase (Thr172, p-AMPK) and total AMPK expression in ventricles of 4-week-old animals (*left*), and quantification of expression (right, n=4). Student's t-test p-value: *, $p<0.05$; **, $p<0.01$.

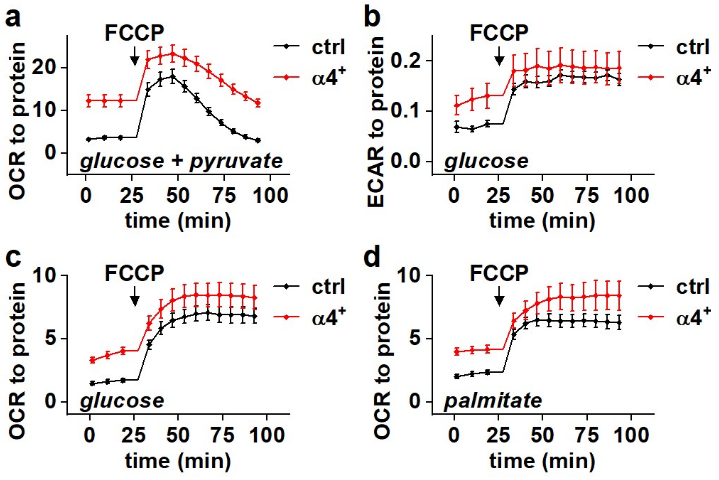

Figure S3. Averaged traces from Seahorse extracellular flux assays. (a) Oxygen consumption rate (OCR) in isolated 4-week-old cardiomyocytes supplemented with glucose and pyruvate (n[ctrl]=5/33 (animals/wells), n[$\alpha 4^{+}$]=5/38). **(b)** Extracellular acidification rate (ECAR) in isolated 4-week-old cardiomyocytes supplemented with glucose (n[ctrl]=5/17, n[$\alpha 4^{+}$]=6/15). **(c)** OCR in isolated 4-week-old cardiomyocytes supplemented with glucose (n[ctrl]=5/25, n[$\alpha 4^{+}$]=6/24). **(d)** OCR in isolated 4-week-old cardiomyocytes supplemented with palmitate (n[ctrl]=5/23, n[$\alpha 4^{+}$]=6/20).

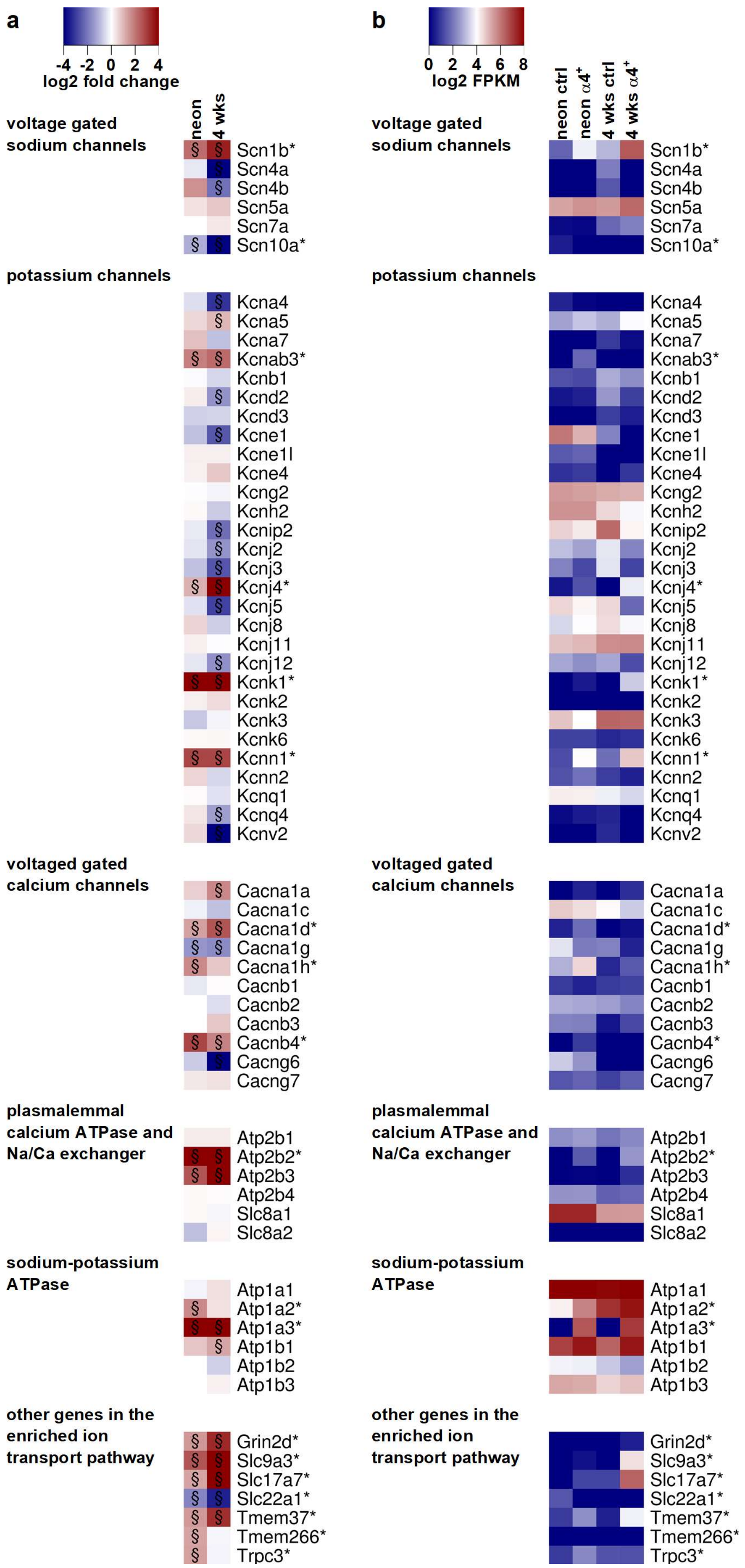
a
-4 -2 0 2 4
log2 fold change
neon
4 wks
voltage gated sodium channels
Scn1b*
Scn4a
Scn4b
Scn5a
Scn7a
Scn10a*
potassium channels
Kcna4
Kcna5
Kcna7
Kcnab3*
Kcnb1
Kcnd2
Kcnd3
Kcne1
Kcne1l
Kcne4
Kcng2
Kcnh2
Kcnip2
Kcnj2
Kcnj3
Kcnj4*
Kcnj5
Kcnj8
Kcnj11
Kcnj12
Kcnk1*
Kcnk2
Kcnk3
Kcnk6
Kcnn1*
Kcnn2
Kcnq1
Kcnq4
Kcnv2
voltaged gated calcium channels
Cacna1a
Cacna1c
Cacna1d*
Cacna1g
Cacna1h*
Cacnb1
Cacnb2
Cacnb3
Cacnb4*
Cacng6
Cacng7
plasmalemmal calcium ATPase and Na/Ca exchanger
Atp2b1
Atp2b2*
Atp2b3
Atp2b4
Slc8a1
Slc8a2
sodium-potassium ATPase
Atp1a1
Atp1a2*
Atp1a3*
Atp1b1
Atp1b2
Atp1b3
other genes in the enriched ion transport pathway
Grin2d*
Slc9a3*
Slc17a7*
Slc22a1*
Tmem37*
Tmem266*
Trpc3*
b
0 2 4 6 8
log2 FPKM
neon ctrl
neon α4+
4 wks ctrl
4 wks α4+
voltage gated sodium channels
potassium channels
voltaged gated calcium channels
plasmalemmal calcium ATPase and Na/Ca exchanger
sodium-potassium ATPase
other genes in the enriched ion transport pathway

Figure S4. Gene expression of cell membrane ion transporters in neonatal and 4-week-old $\alpha4^{+}$ ventricles. **(a)** Heatmap showing transcriptional changes of genes encoding for plasmalemmal ion transporters in $\alpha4^{+}$ ventricles assessed with RNA sequencing. **(b)** Heatmap showing mean FPKM values for experimental groups. §, $p<0.01$ and absolute log2 fold change>1. Asterisk in the gene id marks the genes that were changed in neonatal ventricles belonging to ion transporter pathway (GO biological process).

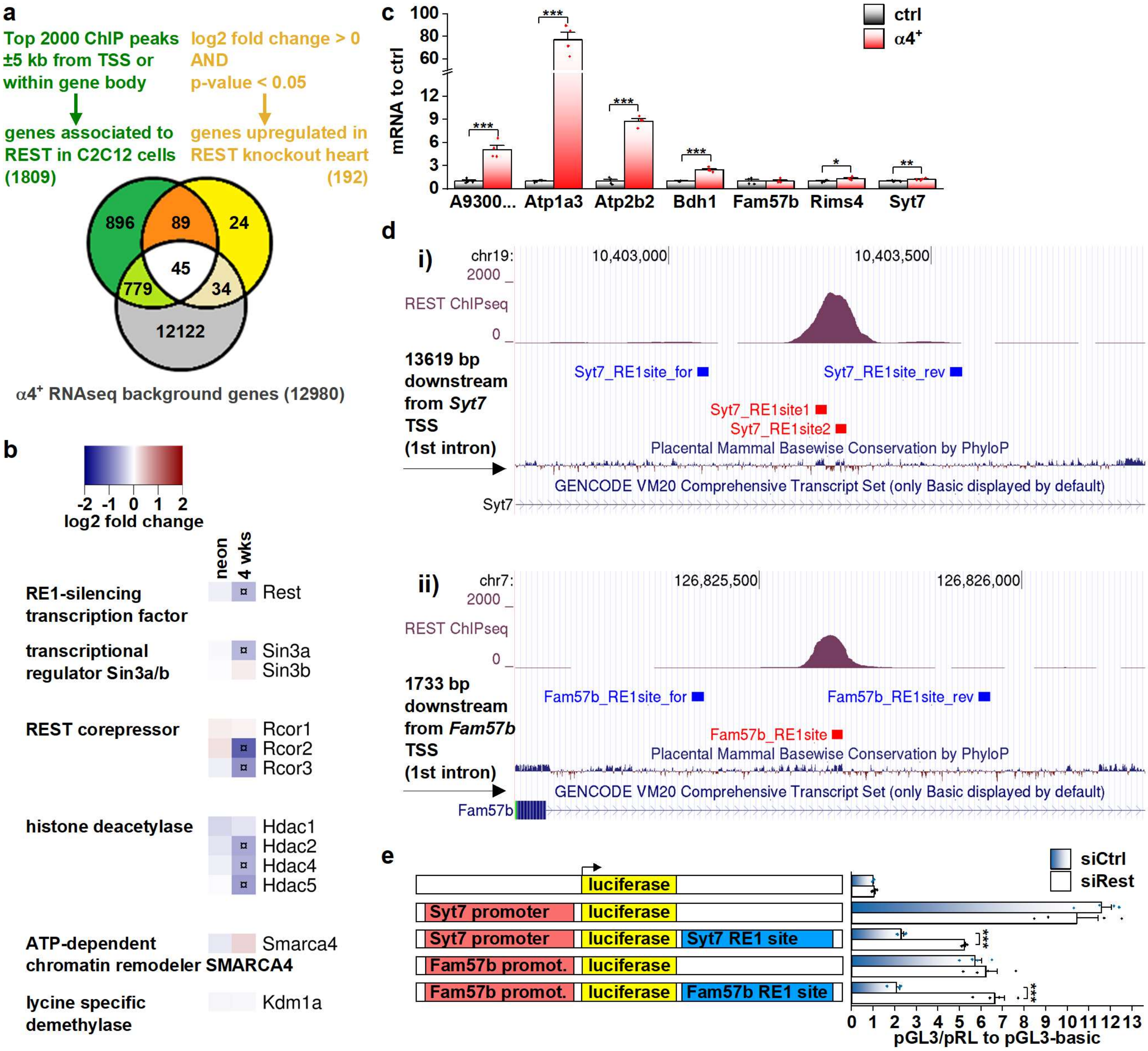

Figure S5. Genes associated with the transcriptional repressor REST are induced by PGC-1α4 in cardiac myocytes. (a) Venn diagram showing the overlap of genes upregulated in REST knockout mouse heart, associated with the REST ChIP sequencing peak in C2C12 cells and passing the expression filter in $\alpha 4^+$ RNA sequencing data. **(b)** Heatmap showing the expression of *Rest* and other REST complex members in $\alpha 4^+$ ventricles assessed from RNA sequencing data. **(c)** Gene expression of Rest target genes in embryonic day 12.5 $\alpha 4^+$ ventricles (n=4). **(d)** Genome tracks (UCSC mm10 browser) from the DNA region surrounding RE1 sites showing ChIP sequencing peaks from C2C12 data, binding sites of primers used for cloning the RE1 site region to luciferase expression plasmid (blue), RE1 consensus sites below ChIP-seq peaks (red) and DNA conservation track from UCSC browser. **i)** *Syt7* RE1 site, **ii)** *Fam57b* RE1 site. **(e)** Effect of Rest siRNA co-transfection on the activity of luciferase constructs containing the RE1 site in HL-1 cells (n=4). Student's t-test p-value: *, $p<0.05$; **, $p<0.01$; ***, $p<0.001$. Heatmap: ¤, $p<0.01$.

Supplementary tables

Table S1. NCBI BioProject identifiers and sample descriptions for RNA sequencing datasets used in Pgc-1α expression analysis.

Dataset	BioProject	Sample description and DOI for publication
DS1	PRJNA 227375	14 weeks old mouse heart 14 days after LAD and SHAM operation (n=4) https://doi.org/10.1093/eurheartj/ehu180
DS2	PRJNA 472253	8 weeks old mouse left ventricles 1 day, 1 week and 8 weeks after LAD and SHAM operation (n=3) https://doi.org/10.1038/s41419-019-2061-8
DS3	PRJNA 470729	11 weeks old mouse heart from TAC (n=2) and SHAM (n=4) operated animals. No publication referring to data available.
DS4	PRJNA 277489	3-4 months old mouse cardiac tissue from TAC and SHAM operated animals 1, 2, 4 and 8 weeks after operation (n=4) https://doi.org/10.1371/journal.pcbi.1004332
DS5	PRJNA 445706	non-failing human left ventricular tissues (32-65 years old) (n=8) https://doi.org/10.1038/s41598-018-32154-2
DS6	PRJNA 291619	human left ventricular biopsies from heart transplantation donors (n=4, 57.5±11.7 years old) and recipients (n=4, 57.5±10.2 years old) suffering from cardiomyopathy https://doi.org/10.5603/CJ.a2017.0052
DS7	PRJNA 280990	human left ventricular tissues from healthy individuals and myotonic dystrophy patients (n=3) https://doi.org/10.1038/ncomms11067
DS8	PRJEB 8360	human left ventricular tissues from dilated cardiomyopathy patients (n=6, samples with deepest sequencing chosen) https://doi.org/10.1371/journal.pone.0097380

Table S2. Boltzmann fitting results of voltage-dependent activation/inactivation of sodium and L-type calcium currents.

		control	$\alpha 4^+$	p-value
**sodium current**				
Activation	$V_{1/2}$	–46.5 ± 1.1	–52.9 ± 0.9	<0.001
	slope	2.86 ± 0,22	1.88 ± 0,27	0.01
Inactivation	$V_{1/2}$	–78.2 ± 0.8	–79.2 ± 0.8	>0.05
	slope	4.68 ± 0.21	4.92 ± 0.34	>0.05
**L-type Ca2+ current**				
Activation	$V_{1/2}$	–2.54 ± 0.48	–7.96 ± 0.73	<0.001
	slope	4.70 ± 0.13	4.02 ± 0.42	>0.05
Inactivation	$V_{1/2}$	–16.60 ± 0.59	–20.04 ± 0.75	<0.001
	slope	5.11 ± 0.22	6.04 ± 0.25	<0.01

Table S3. Top 20 KEGG pathways GO terms from enrichment analysis of differentially expressed genes in neonatal and 4-week-old $\alpha4^+$ ventricles.

KEGG pathway	Count	P-value	Fold enrichment	FDR
**neonatal**				
Bile secretion	8	5.0E–06	11.1	6.0E-03
Neuroactive ligand-receptor interaction	9	9.8E–06	8.2	1.2E–02
Calcium signaling pathway	11	1.9E–05	5.6	2.3E–02
Adrenergic signaling in cardiomyocytes	9	1.0E–03	4.3	1.2E+00
Salivary secretion	6	1.5E–03	6.9	1.8E+00
Pancreatic secretion	6	2.3E–03	6.3	2.6E+00
cAMP signaling pathway	9	2.8E–03	3.6	3.3E+00
Proximal tubule bicarbonate reclamation	4	3.6E–03	12.4	4.2E+00
Gastric acid secretion	5	1.0E–02	5.7	1.2E+01
Insulin secretion	5	1.4E–02	5.3	1.5E+01
cGMP-PKG signaling pathway	7	2.0E–02	3.2	2.2E+01
Cardiac muscle contraction	5	2.7E–02	4.3	2.8E+01
Circadian entrainment	5	2.8E–02	4.2	2.9E+01
Glutamatergic synapse	5	3.6E–02	3.9	3.5E+01
One carbon pool by folate	3	3.9E–02	9.3	3.8E+01
Carbohydrate digestion and absorption	3	5.8E–02	7.5	5.1E+01
Protein digestion and absorption	4	6.0E–02	4.4	5.2E+01
Vascular smooth muscle contraction	5	6.5E–02	3.2	5.5E+01
Mineral absorption	3	6.8E–02	6.9	5.7E+01
Retrograde endocannabinoid signaling	4	8.0E–02	3.9	6.3E+01
**four weeks**				
Neuroactive ligand-receptor interaction	28	1.5E–09	3.5	2.0E–06
ECM-receptor interaction	29	1.8E–09	3.4	2.4E–06
Protein digestion and absorption	24	1.3E–08	3.6	1.7E–05
PI3K-Akt signaling pathway	67	1.7E–08	2.0	2.3E–05
Calcium signaling pathway	32	1.6E–05	2.2	2.1E–02
Malaria	14	7.7E–05	3.4	1.0E–01
Amoebiasis	23	1.1E–04	2.4	1.5E–01
Focal adhesion	42	1.6E–04	1.8	2.1E–01
Cytokine-cytokine receptor interaction	25	2.0E–04	2.2	2.6E–01
Adrenergic signaling in cardiomyocytes	30	3.9E–04	1.9	5.1E–01
Circadian entrainment	20	4.8E–04	2.3	6.2E–01
cGMP-PKG signaling pathway	29	1.6E–03	1.8	2.1E+00
Dilated cardiomyopathy	19	1.7E–03	2.2	2.1E+00
Arginine and proline metabolism	13	3.9E–03	2.5	5.0E+00
Hypertrophic cardiomyopathy (HCM)	17	6.2E–03	2.0	7.8E+00
Ovarian steroidogenesis	9	7.0E–03	2.9	8.8E+00
Histidine metabolism	7	7.8E–03	3.6	9.7E+00
Cholinergic synapse	19	7.9E–03	1.9	9.9E+00
Oxytocin signaling pathway	26	8.3E–03	1.7	1.0E+01
Morphine addiction	14	1.1E–02	2.1	1.4E+01

Gene ontology term - Biological process	Count	P-value	Fold enrichment	FDR
**neonatal**				
ion transport	19	9.7E–07	4.0	1.6E–03
regulation of ion transmembrane transport	8	4.1E–05	8.4	6.6E–02
transport	40	7.3E–05	1.9	1.2E–01
transmembrane transport	13	7.7E–05	4.1	1.2E–01
regulation of atrial cardiac muscle cell membrane depolarization	4	8.8E–05	39.8	1.4E–01
calcium ion transmembrane transport	7	1.1E–04	9.1	1.7E–01
neurotransmitter transport	5	1.2E–04	18.6	1.9E–01
G-protein coupled receptor signaling pathway	12	2.1E–04	4.0	3.3E–01
potassium ion transport	7	2.4E–04	7.9	3.8E–01
cardiac conduction	4	3.5E–04	26.5	5.7E–01
sensory perception of sound	7	4.3E–04	7.1	6.8E–01
sodium ion transport	6	1.2E–03	7.5	1.8E+00
sodium ion transmembrane transport	4	1.4E–03	17.0	2.3E+00
visual learning	5	4.7E–03	7.3	7.2E+00
regulation of membrane potential	5	4.7E–03	7.3	7.2E+00
locomotion	3	5.5E–03	25.6	8.4E+00
one-carbon metabolic process	4	5.5E–03	10.8	8.5E+00
signal transduction	18	6.0E–03	2.1	9.2E+00
adult walking behavior	4	7.1E–03	9.9	1.1E+01
calcium ion-regulated exocytosis of neurotransmitter	3	7.2E–03	22.4	1.1E+01
**four weeks**				
cell adhesion	81	6.2E–13	2.3	1.2E–09
regulation of ion transmembrane transport	30	3.3E–12	4.1	6.1E–09
G-protein coupled receptor signaling pathway	58	1.6E–11	2.5	3.0E–08
ion transport	72	5.0E–09	2.0	9.4E–06
potassium ion transport	23	1.5E–07	3.4	2.8E–04
extracellular matrix organization	28	1.2E–06	2.7	2.2E–03
collagen fibril organization	16	1.4E–06	4.1	2.6E–03
response to drug	56	1.5E–06	1.9	2.9E–03
positive regulation of cell migration	44	1.9E–06	2.1	3.6E–03
signal transduction	102	5.4E–06	1.5	1.0E–02
ossification	23	8.6E–06	2.8	1.6E–02
sodium ion transport	19	1.1E–05	3.1	2.1E–02
cell-cell signaling	15	2.1E–05	3.6	3.9E–02
inflammatory response	41	2.8E–05	2.0	5.2E–02
positive regulation of cell proliferation	65	3.0E–05	1.7	5.6E–02
positive regulation of cell-substrate adhesion	14	3.1E–05	3.7	5.9E–02
endodermal cell differentiation	11	3.3E–05	4.6	6.2E–02
cellular response to interleukin-1	17	5.3E–05	3.0	1.0E–01
positive regulation of Rho protein signal transduction	9	9.8E–05	5.1	1.8E–01
negative regulation of Wnt signaling pathway	16	1.2E–04	3.0	2.2E–01

Gene ontology term - Cellular compartment	Count	P-value	Fold enrichment	FDR
**neonatal**				
synapse	21	1.5E–08	4.8	1.9E–05
integral component of membrane	81	1.6E–08	1.8	2.0E–05
plasma membrane	67	2.0E–08	1.9	2.5E–05
integral component of plasma membrane	21	1.2E–05	3.1	1.5E–02
membrane	100	1.0E–04	1.3	1.3E–01
synaptic vesicle membrane	6	1.7E–04	11.2	2.1E–01
extracellular region	24	8.7E–04	2.1	1.1E+00
voltage-gated calcium channel complex	4	1.4E–03	17.1	1.8E+00
cell junction	17	1.8E–03	2.4	2.2E+00
anchored component of membrane	6	4.8E–03	5.4	5.8E+00
basement membrane	6	8.6E–03	4.7	1.0E+01
dendrite	12	1.0E–02	2.4	1.2E+01
apical plasma membrane	9	1.2E–02	2.9	1.4E+01
apical dendrite	3	1.9E–02	13.8	2.2E+01
brush border membrane	4	2.0E–02	6.8	2.3E+01
neuronal cell body	12	2.6E–02	2.1	2.8E+01
sarcolemma	6	3.0E–02	3.4	3.2E+01
extracellular space	18	3.4E–02	1.7	3.6E+01
cytoplasmic vesicle	14	3.7E–02	1.9	3.8E+01
axon terminus	4	4.1E–02	5.2	4.1E+01
**four weeks**				
extracellular region	259	4.1E–68	3.0	6.0E–65
extracellular space	194	2.2E–34	2.4	3.2E–31
proteinaceous extracellular matrix	85	1.3E–28	3.6	2.0E–25
extracellular matrix	77	1.1E–16	2.7	1.7E–13
plasma membrane	368	1.3E–13	1.4	1.9E–10
collagen trimer	29	7.2E–13	4.5	1.1E–09
integral component of plasma membrane	102	5.5E–12	2.0	8.1E–09
integral component of membrane	441	2.3E–09	1.3	3.4E–06
basement membrane	30	1.7E–08	3.1	2.5E–05
cell surface	81	3.6E–08	1.8	5.4E–05
neuronal cell body	77	3.5E–07	1.8	5.1E–04
perikaryon	25	3.6E–05	2.4	5.3E–02
anchored component of membrane	22	4.2E–05	2.6	6.1E–02
synapse	57	6.9E–05	1.7	1.0E–01
blood microparticle	19	2.1E–04	2.6	3.0E–01
axon	49	4.1E–04	1.7	6.0E–01
asymmetric synapse	7	5.6E–04	5.5	8.3E–01
perinuclear region of cytoplasm	95	1.6E–03	1.4	2.3E+00
dendrite	56	2.0E–03	1.5	2.9E+00
extracellular exosome	285	2.8E–03	1.2	4.0E+00

Gene ontology term - Molecular function	Count	P-value	Fold enrichment	FDR
**neonatal**				
G-protein coupled receptor activity	12	1.4E–06	6.7	2.0E–03
voltage-gated ion channel activity	8	4.8E–05	8.2	6.6E–02
ion channel activity	6	3.2E–03	5.9	4.4E+00
transporter activity	7	3.4E–03	4.7	4.7E+00
calcium channel activity	5	3.8E–03	7.7	5.2E+00
voltage-gated calcium channel activity	4	3.9E–03	12	5.2E+00
signal transducer activity	10	7.5E–03	2.9	9.9E+00
sodium channel activity	3	1.2E–02	17	1.5E+01
aldehyde dehydrogenase (NAD) activity	3	2.0E–02	13	2.4E+01
oxidoreductase activity, acting on the aldehyde or oxo group of donors, NAD or NADP as acceptor	3	3.3E–02	10	3.7E+01
low voltage-gated calcium channel activity	2	3.4E–02	58	3.8E+01
voltage-gated calcium channel activity involved SA node cell action potential	2	3.4E–02	58	3.8E+01
formyltetrahydrofolate dehydrogenase activity	2	3.4E–02	58	3.8E+01
symporter activity	4	3.9E–02	5.3	4.2E+01
insulin-like growth factor binding	3	4.5E–02	8.7	4.7E+01
PDZ domain binding	5	5.3E–02	3.5	5.3E+01
calmodulin binding	6	5.4E–02	2.9	5.4E+01
transmembrane transporter activity	3	5.8E–02	7.6	5.6E+01
hydroxymethyl-, formyl- and related transferase activity	2	6.6E–02	29	6.2E+01
4-trimethylammoniobutyraldehyde dehydrogenase activity	2	6.6E–02	29	6.2E+01
**four weeks**				
G-protein coupled receptor activity	42	7.4E–12	3.1	1.2E–08
voltage-gated ion channel activity	29	3.1E–11	4.0	5.1E–08
calcium ion binding	85	4.4E–10	2.0	7.3E–07
heparin binding	34	5.2E–09	3.0	8.6E–06
cytokine activity	26	1.6E–07	3.1	2.7E–04
scavenger receptor activity	15	3.6E–07	4.7	5.8E–04
extracellular matrix structural constituent	15	6.8E–07	4.5	1.1E–03
growth factor activity	26	1.2E–06	2.8	1.9E–03
signal transducer activity	51	2.2E–06	2.0	3.7E–03
fibronectin binding	14	2.9E–06	4.3	4.7E–03
hormone activity	15	1.0E–05	3.8	1.6E–02
voltage-gated potassium channel activity	12	2.8E–05	4.2	4.6E–02
peptidase inhibitor activity	16	9.7E–05	3.0	1.6E–01
extracellular matrix binding	12	1.2E–04	3.7	2.0E–01
serine-type endopeptidase inhibitor activity	14	1.7E–04	3.2	2.8E–01
metalloendopeptidase activity	19	2.9E–04	2.5	4.8E–01
insulin-like growth factor binding	10	4.2E–04	3.9	6.8E–01
sodium channel activity	7	5.9E–04	5.4	9.7E–01
metallopeptidase activity	23	8.6E–04	2.1	1.4E+00
ion channel activity	18	8.9E–04	2.4	1.5E+00

Table S4. List of REST target genes that were upregulated in $\alpha4^{+}$ ventricles and ranking order of their expression in different mouse tissues. Expression data from ENCODE project RNA sequencing analysis downloaded from NCBI gene database.

CNS E11.5	CNS E14	CNS E18	large intestine adult	adrenal adult	bladder adult	cerebellum adult	colon adult	cortex adult	duodenum adult	frontal lobe adult	genital fat pad adult	heart adult	kidney adult	limb E14.5	liver E14	liver E14.5	liver E18	liver adult	lung adult	mammary gland adult	ovary adult	placenta adult	small intestine adult	spleen adult	stomach adult	subcutaneous fat pad adult	testis adult	thymus adult	whole brain E14.5	gene
**Rest targets upregulated in neonatal and 4-week-old α4+ ventricles**																														
13	6	10	23	8	24	1	15	4	9	2	11	3	12	22	21	27	25	27	17	27	7	19	20	26	27	16	14	18	5	A930029G22Rik
8	6	4	18	7	26	1	20	3	16	2	17	22	21	24	27	25	23	30	13	10	9	28	15	11	19	12	29	14	5	Atp1a3
9	6	4	20	11	22	2	19	1	24	3	26	18	16	29	17	15	12	14	10	28	7	30	21	27	25	8	13	23	5	Atp2b2
20	14	10	4	30	6	15	5	18	7	17	23	19	3	21	9	8	2	1	28	27	24	22	12	26	11	29	25	16	13	Bdh1
10	4	2	21	8	22	3	19	6	17	5	11	13	18	24	30	28	25	29	12	7	14	26	20	27	16	9	15	23	1	Fam57b
14	8	6	25	1	29	3	9	4	19	5	15	16	23	20	18	17	12	30	22	11	2	28	21	26	23	10	13	27	7	Rims4
10	6	4	15	11	24	3	8	1	20	2	22	9	19	18	28	25	30	27	12	21	7	26	16	29	14	17	13	23	5	Syt7
**Rest targets upregulated in 4-week-old α4+ ventricles**																														
8	7	4	22	11	15	1	16	2	14	3	18	9	28	16	10	12	26	30	25	13	23	29	24	20	19	21	5	27	6	Acsl6
8	6	4	19	15	23	1	10	2	13	3	18	25	24	11	30	29	28	27	22	16	7	26	14	21	17	12	9	20	5	Cacna1a
5	4	3	16	12	23	1	8	7	19	6	18	9	25	10	28	27	29	30	22	24	13	21	17	20	14	26	11	15	2	Cacna2d2
no expression data available																														Gm13054
7	4	5	21	12	8	6	14	1	18	2	17	13	23	20	28	27	29	30	9	22	11	24	19	25	15	16	10	26	3	Gnao1
24	13	8	14	5	23	1	4	3	7	2	20	6	21	26	30	28	29	27	16	17	9	15	10	22	11	19	25	18	12	Hcn2
6	3	2	20	15	10	4	18	7	25	5	8	17	21	9	28	26	29	30	16	19	11	12	24	27	22	14	13	23	1	Map1b
4	2	3	21	9	11	5	14	8	20	7	22	24	25	16	29	28	26	30	18	12	6	23	19	15	17	13	27	9	1	Meis3
6	3	2	22	11	19	7	17	5	21	4	24	14	13	10	27	20	28	30	18	16	15	23	25	29	12	26	8	9	1	Mgat5b
3	7	6	22	16	17	9	30	10	26	5	23	1	12	14	29	27	25	24	4	19	12	15	28	20	21	18	11	2	8	Nppa
2	6	5	17	14	9	8	20	15	20	10	20	1	16	7	20	20	19	20	11	20	20	3	20	20	20	18	13	4	12	Nppb
9	7	4	11	10	20	3	8	1	13	2	14	25	19	18	23	24	29	30	15	27	22	26	12	16	6	28	21	17	5	Ptprn

Table S5. Sequences of primers and probes used in gene expression analysis. Genes with probe sequence were analyzed with Taqman based chemistry and genes without probe sequence were analyzed with Sybr Green chemistry.

Short name	Name, Gene ID		Nucleotide sequence
18s	18S ribosomal RNA	5'	TGGTTGCAAAGCTGAAACTTAAAG
		3'	AGTCAAATTAAGCCGCAGGC
	19791	probe	CCTGGTGGTGCCCTTCCGTCA
B2m	beta-2 microglobulin	5'	GCCTGTATGCTATCCAGA
		3'	GAAGGACATATCTGACATCTC
	12010	probe	TATACTCACGCCACCCACCG
Pgc-1α	peroxisome proliferator activated receptor,	5' (V)	AGCGACCAATCGGAAATCAT
(total	gamma,coactivator 1 alpha	3' (VI)	GCAAGTTTGCCTCATTCTCTTCA
Pgc-1α)	19017	probe	TCCAACCAGTACAACAATGAGCCTGCG
Pgc-1α	peroxisome proliferator activated receptor,	5' (III)	CAGAGTGGATTGGAGTTG
(exon 1a)	gamma,coactivator 1 alpha	3' (IV)	GGTCAAGTTCAGGAAGATC
	19017	probe	TCACCAACCAGAGCAGCACA
Pgc-1α	peroxisome proliferator activated receptor,	5' (I)	AGCAGAATGAGTGACATG
(exon 1b +	gamma,coactivator 1 alpha	3' (IV)	GGTCAAGTTCAGGAAGATC
exon 1b')	19017	probe	TCACCAACCAGAGCAGCACA
Pgc-1α	peroxisome proliferator activated receptor,	5' (II)	CACCTCAGACCCACTATG
(exon 1b')	gamma,coactivator 1 alpha	3' (IV)	GGTCAAGTTCAGGAAGATC
	19017	probe	TCACCAACCAGAGCAGCACA
Pgc-1α	peroxisome proliferator activated receptor,	5' (VII)	TCACACCAAACCCACAGAAA
(exon 7b)	gamma,coactivator 1 alpha	3' (VIII)	GTCACTGGAAGATATGGCACAT
	19017	probe	CTGGGGTCAGAGGAAGAGATAAAGTTGTT
Pgc-1α	peroxisome proliferator activated receptor,	5' (III)	CAGAGTGGATTGGAGTTG
(exon 1a	gamma,coactivator 1 alpha	3' (VIII)	GTCACTGGAAGATATGGCACAT
to exon 7b)	19017		

Pgc-1α (exon 1b/1b' to exon 7b)	peroxisome proliferator activated receptor, gamma,coactivator 1 alpha<br>19017	5' (I)<br>3' (VIII)	AGCAGAATGAGTGACATG<br>GTCACTGGAAGATATGGCACAT
α-Mhc	myosin, heavy polypeptide 6, cardiac muscle, alpha<br>17888	5'<br>3'<br>probe	GGTGCCAAGAAGATGCACG<br>TTATGTTTATTGTGTATTGGCCACAG<br>CGAGGAATAACCTCTCCAGCAGACCCTC
β-Mhc	myosin, heavy polypeptide 7, cardiac muscle, beta<br>140781	5'<br>3'<br>probe	AGCTCTAAGGGTGCCCGTG<br>TGCTTCCACCTAAAGGGCTG<br>AGCCCTCAGACCTGGAGCCTTTGC
Anp	natriuretic peptide type A<br>230899	5'<br>3'<br>probe	GAAAAGCAAACTGAGGGCTCTG<br>CCTACCCCCGAAGCAGCT<br>TCGCTGGCCCTCGGAGCCT
Bnp	natriuretic peptide type B<br>18158	5'<br>3'<br>probe	AGGCGAGACAAGGGAGAACA<br>GGAGATCCATGCCGCAGA<br>CATCATTGCCTGGCCCATCGC
Tfam	transcription factor A, mitochondrial<br>21780	5'<br>3'<br>probe	TTCGTTACGACAATGAAATGAAGTC<br>TCGACGGATGAGATCACTTCG<br>TGGGAAGAGCAGATGGCTGAAGTTGG
Cpt1b	carnitine palmitoyltransferase 1b, muscle<br>12895	5'<br>3'<br>probe	GACAGAAGCAAACCTGAG<br>GTCACAGTGAACTGGAAA<br>CTACTGCCTGGTGTGCTTCC
Sod2	superoxide dismutase 2, mitochondrial<br>20656	5'<br>3'<br>probe	GAGATGTTACAACTCAGG<br>GCTTCTCCTTAAACTTCTC<br>TGATAGCCTCCAGCAACTCTCC
Ucp3	uncoupling protein 3 (mitochondrial, proton carrier)<br>22229	5'<br>3'<br>probe	TTGCCTCCATTCGAATTG<br>GCGTATCATGGCTTGAAA<br>CCTCTACGACTCTGTCAAGCAGTT

Pparα	peroxisome proliferator activated receptor<br>alpha<br>19031	5'<br>3'<br>probe	ACGATGCTGTCCTCCTTGATG<br>GTGTGATAAAGCCATTGCCGT<br>ACAAAGACGGGATGCTGATCGCG
Pparγ	peroxisome proliferator activated receptor<br>gamma<br>19016	5'<br>3'<br>probe	AGTGGAGACCGCCCAGG<br>GCAGCAGGTTGTCTTGGATGT<br>TTGCTGAACGTGAAGCCCATCGAG
Abat	4-aminobutyrate aminotransferase<br><br>268860	5'<br>3'	CCTCTTCATACCTGGATC<br>CCTCTGCTCTCTTCATAG
Acads	acyl-Coenzyme A dehydrogenase, short chain<br><br>11409	5'<br>3'	GGACCCATTCTGAAGTTTG<br>AGGAGTTGGTGATCCAAG
Aldh5a1	aldhehyde dehydrogenase family 5,<br>subfamily A1<br>214579	5'<br>3'	GGTACGACTTAATGATACAA<br>TTGGCAGAGGTATAGATG
Atp1b1	ATPase, Na+/K+ transporting,<br>beta 1 polypeptide<br>11931	5'<br>3'	CCAAGTAATGCTGCTAAC<br>CTCAGTCTTCTGGATCTG
Cacna1h	calcium channel, voltage-dependent,<br>T type, alpha 1H subunit<br>58226	5'<br>3'	CTCCGACTGGTTTGTAAC<br>CACTCAACATCCTCACAG
Ckmt2	creatine kinase, mitochondrial 2<br><br>76722	5'<br>3'	GCATCCAAGATTACTCAC<br>CTCAGACAGCTTGTAGTA
Cox6a2	cytochrome c oxidase subunit 6A2<br><br>12862	5'<br>3'	GCCTCTAAAGGTCCTGAG<br>TGGTGATACGGGATGAAC

Cox7a1	cytochrome c oxidase subunit 7A1<br><br>12865	5' CTGAGGACGCAAAATGAG<br>3' CGTCATGGTCAGTCTGTA
Cox8b	cytochrome c oxidase subunit 8B<br><br>12869	5' CGAAGTTCACAGTGGTTC<br>3' ACGACTATGGCTGAGATC
Dgat1	diacylglycerol O-acyltransferase 1<br><br>13350	5' GATGCTGATCCTGAGTAA<br>3' GTCCTTCAGAAACAGAGA
Epas1	endothelial PAS domain protein 1<br><br>13819	5' CAGTCTGCTCTGAAAATG<br>3' CAAGGATGAGTGAAGTCA
Fblim1	filamin binding LIM protein 1<br><br>74202	5' GACCAATGCCTCACTCTC<br>3' CGACTTCCCTGGTAAGAC
Fndc5	fibronectin type III domain containing 5<br><br>384061	5' GGATGAAGTGGTCATTGG<br>3' TCACCTCCTGAATGAACC
Glrx	glutaredoxin<br><br>93692	5' GGTCTTCTGGAGTTTGTG<br>3' GTCTGTTGCATGGAGATTA
Gpr162	G protein-coupled receptor 162<br><br>14788	5' GTGGTGAGCTTCTTCTCC<br>3' CTCACAGGACCAGATGAA
Mpc2	mitochondrial pyruvate carrier 2<br><br>70456	5' GCTGCCAAAGAAATTGAG<br>3' CTGTGCTGAGTTTCTCTG

Mtfp1	mitochondrial fission process 1<br><br>67900	5' TGGTGTGGTTGAGCTATG<br>3' AGACGGTTGATGGTGAAG
Ndufa6	NADH:ubiquinone oxidoreductase subunit A6<br><br>67130	5' GGTGAAGCCCATTTTCAG<br>3' GTGACATGGGCATTCTTC
Pcyt2	phosphate cytidylyltransferase 2, ethanolamine<br><br>68671	5' CTGTGACTTCTGTGTTCA<br>3' CTGCTTCACTTCCTCATA
Phyh	phytanoyl-CoA hydroxylase<br><br>16922	5' CCTGAACAATTCCAGTATA<br>3' GCAGATTCTTTCAAACTC
S100a1	S100 calcium binding protein A1<br><br>20193	5' GAGCAAGAAAGAACTGAA<br>3' CCAGAAGAAGTTGTTACAA
Shmt1	serine hydroxymethyltransferase 1<br><br>20425	5' GGAACCTTTTCACAAAATC<br>3' CGACAACATCTTCTCATG
Slc4a3	solute carrier family 4 (anion exchanger), member 3<br>20536	5' CACTGACTATCACGAATTAGG<br>3' GGGAATCACAATGCTACC
Slc25a39	solute carrier family 25, member 39<br><br>68066	5' GAGTCTCTCCTACACCAA<br>3' GCACAATCTTCACAAAGG
Slc12a7	solute carrier family 12, member 7<br><br>20499	5' CCAACTATACCAACCTGA<br>3' GAGGAAGGACTCCATAAC

Atp1a1	ATPase, Na+/K+ transporting,<br>alpha 1 polypeptide<br>11928	5'<br>3'<br>probe	TGCTCTCTTCTCTTTCTAGTC<br>GTCACCATGCTCCGATAC<br>CAGGCTCATACTTGTCTCGTCCA
Atp1a2	ATPase, Na+/K+ transporting,<br>alpha 2 polypeptide<br>98660	5'<br>3'<br>probe	CGAACCATCCAACGATAA<br>CACCATGTTCTTGAAGGA<br>TAGCGGCTGTAGTTATCGTCACC
Atp1a3	ATPase, Na+/K+ transporting,<br>alpha 3 polypeptide<br>232975	5'<br>3'<br>probe	CTGGATGACCTCAAGAAG<br>CACGCAGTCAGTATTGTA<br>ACCTCTTCTACTGACATCTTGTGCTC
A930029<br>G22Rik	RIKEN cDNA A930029G22 gene<br><br>654806	5'<br>3'	GGGAAGCCAGACAGAAAG<br>TTGCCAGAAGCACAGAAA
Atp2b2	ATPase, Ca++ transporting,<br>plasma membrane 2<br>11941	5'<br>3'<br>probe	TCGCCTGTTGAAGGTTTA<br>GGTGGTAGAAGGACAGTC<br>CAGGCACTGCTCCAGACTTG
Bdh1	3-hydroxybutyrate dehydrogenase, type 1<br><br>71911	5'<br>3'<br>probe	AAGGCTTCCTTGTATTTG<br>CCTTCTCAGGATCTTTCA<br>ATCGTCTCCACCGCCTTCTC
Fam57b	family with sequence similarity 57 member B<br><br>68952	5'<br>3'<br>probe	CTACCTGCACAAGGAGTTC<br>GCAGCCTAGAAAGAAATCTC<br>TTGTCGCCACACCACTGAGA
Rims4	regulating synaptic membrane exocytosis 4<br><br>241770	5'<br>3'	GCATGAACTCCTTCGATG<br>GTCTCTGTGCTTCTCTGG
Syt7	synaptotagmin VII<br><br>54525	5'<br>3'<br>probe	CCACACGATGAGTCTGAC<br>GTAGCCAACACTGAACTG<br>CTCTGTCTCGGACCTCGTCAAC

Cacna1a	calcium channel, voltage-dependent,<br>P/Q type, alpha 1A subunit<br>12286	5'<br>3'	GGAACCATACTTCATTGGAA<br>TGTCCGTAGGTCAAACTC
Hcn2	hyperpolarization-activated,<br>cyclic nucleotide-gated K+ 2<br>15166	5'<br>3'<br>probe	CTGATCAGCATGATGCTACTGCT<br>GCATGGGCACCAGGAACT<br>TGCCACTGGGACGGTTGCCTG
Meis3	Meis homeobox 3<br><br>17537	5'<br>3'	GGATCTTTCCCAAAGTGG<br>TGCGATTAGACTGGTCAA
Mgat5b	mannoside acetylglucosaminyltransferase 5,<br>isoenzyme B<br>268510	5'<br>3'	CTGGGAGAGATGGTACAG<br>CGTGATAGTCCGTGTAGA
Ptprn	protein tyrosine phosphatase, receptor type, N<br><br>19275	5'<br>3'	CCTCTTACAAGTCACTTCC<br>CTGGGTAAGGTCATCATG
Cdkn1a	cyclin-dependent kinase inhibitor 1A (P21)<br><br>12575	5'<br>3'<br>probe	CCAGCCTGACAGATTTCTA<br>CACAGAGTGAGGGCTAAG<br>CCTCCTGACCCACAGCAGAA

Table S6. Sequences of primers used in luciferase plasmid cloning.

Cloned site		Nucleotide sequence
Fam57b promoter	forward	AATACGCGTTTCTGAGGACCAGACGAGGT
	reverse	AAACTCGAGCCAGGTCCACAGCTACACAG
Syt7 promoter	forward	AATACGCGTGGAAAATCTGGGGTGGATCT
	reverse	AAACTCGAGATGGTCCCCTTCGTCGCC
Fam57b RE1 site	forward	AATGTCGACATCGCTCCTACTCTGTTCCTATC
	reverse	AAAGTCGACGTTCTAGGCACTTAACACACTCA
Syt7 RE1 site	forward	AATGTCGACTGTATTGGCGGAGAGACTTGT
	reverse	AAAGTCGACGGAAAGAGGTAGTTAGAGAGGGA

Supplementary methods

Cardiomyocyte isolation and culture

Adult mouse ventricular myocytes at the age of four or 18 weeks were obtained by enzymatic dissociation as previously described[1] (AfCS Procedure Protocol PP00000125). Ten minutes after heparin injection mice were sacrificed by cervical dislocation and the heart was removed. The aorta was cannulated and the heart was placed in a Langendorff apparatus for retrogrde perfusion (37°C, 3 mL/min) in adult cardiomyocyte buffer (113 mM NaCl, 4.7 mM KCl, 0.6 mM KH_2PO_4, 0.6 mM NaH_2PO_4, 1.2 mM $MgCl_2$, 32 µM Phenol red, 20.5 mM $NaHCO_3$, 10 mM $KHCO_3$, 10 mM HEPES, 30 mM taurine, 5.5 mM glucose and 10 mM butanedione monoxime (BDM)). The buffer was supplemented with 140 µg/mL porcine trypsin (Sigma-Aldrich, St Louis, MO, USA), 25 µg/mL liberase (Roche Applied Science, Mannheim, Germany) and 12.5 µM $CaCl_2$ to break down the extracellular matrix. After removing the heart from perfusion, the ventricles were cut into small pieces and single cells were gently dissociated by pipetting the tissue pieces with a glass Pasteur pipette in the enzyme solution. Inactivation of the enzymes was achieved by addition of 5 % fetal bovine serum (FBS, Gibco, ThermoFisher Scientific, Waltham, MA, USA), after which the suspension was centrifuged (100 g, 1 min) and the cell pellet suspended into adult cardiomyocyte buffer supplemented with 10 % FBS and 12.5 µM $CaCl_2$. The Ca^{2+} concentration was then gradually increased to 1 mM. Isolated cardiomyocytes were plated on glass coverslips or cell culture dishes coated with mouse laminin (Sigma-Aldrich, St Louis, MO, USA). For metabolic analysis, cells were plated on XF24 Cell Culture Microplates (Agilent Technologies, Santa Clara, CA, USA) coated with matrigel (BD Matrigel matrix, growth factor reduced, BD Biosciences, Franklin Lakes, NJ, USA) diluted 1:1 in XF Assay medium (Agilent Technologies, Santa Clara, CA, USA) (pH 7.4). For calcium imaging and electrophysiological recordings cells were plated in adult cardiomyocyte buffer supplemented with 10 % FBS and 1 mM $CaCl_2$, incubated at 37 °C in 5 % CO2 and used within four hours after isolation. For 24-hour adenoviral transduction, cells were plated in adult cardiomyocyte plating medium (M199 medium (Gibco, ThermoFisher Scientific, Waltham, MA, USA) supplemented with 5 % FBS, 10 mM BDM and 1 % penicillin-streptomycin (PS, Gibco, ThermoFisher Scientific, Waltham, MA, USA)), and three hours later the solution was changed to adult cardiomyocyte culture medium (M199 medium supplemented with 2 mg/mL BSA (Sigma-Aldrich, St Louis, MO, USA), 2 mM L-carnitine (Sigma-Aldrich, St Louis, MO, USA), 5 mM creatine (Sigma-Aldrich, St Louis, MO, USA), 5 mM taurine (Sigma-Aldrich, St Louis, MO, USA), 1.3 mM L-glutamine (Gibco, ThermoFisher Scientific, Waltham, MA, USA), 0.1 µM insulin (Sigma-Aldrich, St Louis, MO, USA), 0.1 nM triiodothyronine (Sigma-Aldrich, St Louis, MO, USA), 2.5 mM pyruvate (Gibco, ThermoFisher Scientific, Waltham, MA, USA) and 1 % PS). For metabolic analysis, cells were suspended into XF Assay medium and incubated at 37 °C in an atmospheric incubator for one hour before the assay.

Neonatal ventricular cardiomyocytes were isolated 1-2 days after birth as previously[1]. The animal was sacrificed by decapitation, the heart was removed, and the ventricles were cut into small pieces. Ventricle pieces were incubated for 1.5 hours in neonatal cardiomyocyte buffer (100 mM NaCl, 1.2 mM KH_2PO_4, 4 mM $MgSO_4 \bullet 6H_2O$, 10 mM HEPES, 50 mM taurine, 20 mM glucose and 10 mM KCl, pH 6.9) supplemented with 2 mg/mL collagenase type II (Worthington, Lakewood, NJ, USA), 2 mg/mL pancreatine (Sigma-Aldrich, St Louis, MO, USA) and 1 % PS. The solution with tissue pieces was pipetted with a plastic pipette and dissociated cells were centrifuged for 5 minutes at 200 g. The supernatant was discarded, and the cells plated on dishes coated with 1.25 µg/mL fibronectin in 0.02 % gelatin solution in neonatal cardiomyocyte culture medium (Dulbecco's Modified Eagle Medium plus GlutaMAX I (Gibco, ThermoFisher Scientific, Waltham, MA, USA) supplemented with 10 % FBS and 1 % PS).

Confocal calcium imaging

Cardiomyocytes were loaded with Fluo-4-acetoxymethyl-ester (10 µM; 0.02% pluronic acid, Invitrogen, ThermoFisher Scientific, Waltham, MA, USA) in DMEM in a humified incubator at 37 °C in 5 % CO2 for 20 minutes. Cells were then placed in a recording chamber (Cell MicroControls, Norfolk, VA, USA, flow rate 1-2 mL/min, chamber volume 0.4 mL), where they were continuously perfused with DMEM that was oxygenated with carbogen gas. Measurements were carried out after an incubation period of 20 min to allow de-esterification of the dye. All experiments were carried out at 37 °C sustained by a temperature controller (TC2BIP, Cell MicroControls, Norfolk, VA, USA). Only quiescent, rod-shaped cells showing clear cross-striations were used for recordings. Measurements were performed with a FluoView 1000 confocal inverted microscope (Olympus,

Tokyo, Japan) as previously[1]. Cells were excited at 488 nm and the emitted light was collected at 500-600 nm through a 40X or 60X objective lens in line-scan mode. For cardiomyocyte excitation, myocytes were stimulated with voltage pulses (1 ms) 50% over the excitation threshold through two platinum wires located on both sides of the chamber. To assess sarcoplasmic reticulum calcium content, 10 mM caffeine (Sigma-Aldrich, St Louis, MO, USA) was applied directly to the studied area with a local perfusion manifold (Cell MicroControls, Norfolk, VA, USA). FluoView software was used for image analysis. Fluo-4 fluorescence intensity is measured as F/F0 - ratio, where F is the background subtracted fluorescence intensity and F0 is the background-subtracted minimum fluorescence value measured from each cell at rest. Fluorescence line-scan images were used to measure cardiomyocyte shortening in adult cardiomyocytes. To reveal the movement of myocyte edges, fluorescence images were processed to remove all fluorescence except background.

Patch-clamp recordings

Coverslips with attached cells were transferred to the recording chamber where they were perfused with DMEM or Tyrode solution (146 mM, NaCl, 4.5 mM KCl, 1.1 mM $CaCl_2$, 1 mM $MgCl_2$, 10 mM HEPES, 10 mM glucose, pH 7.4 adjusted with NaOH). All HEPES-buffered solutions were continuously bubbled with 100% oxygen. Carbogen gas was used for DMEM. An Axopatch 200B patch-clamp amplifier in combination with a Digidata 1440A and Clampex 10 software (Molecular Devices Inc. , Sunnyvale, CA, USA) were used for the whole cell currents and action potential (AP) recordings as previously[1]. Ag/AgCl electrodes (World Precision Instruments Inc., Sarasota, FL, USA) were used for recording and bath electrodes. Patch pipettes were pulled from borosilicate glass capillary tubing (1.5 mm OD, 0.86 mm ID, Harvard Apparatus, Edenbridge, UK) with a micropipette puller (Sutter P-97, Sutter Instrument Company, Novato, CA, USA) and fire-polished. Patch electrode resistances were 1.5-3 MΩ when filled with any pipette solution. The junction potential was corrected by setting the baseline of the test potential as zero before breaking the cell membrane. After establishment of a ruptured patch, the Tyrode solution was changed to the recording solution depending on the current in question. Recordings were carried out after a period of 5 min to allow adequate intracellular dialysis. The cell capacitance and series resistance were compensated electronically, monitored throughout the experiments, and corrected for time-dependent changes. Recordings with an unstable or poor access resistance (10% change over the period of recording or initial access resistance larger than 10 MΩ) were discarded. Typically, the access resistance was 3-6 MΩ. In voltage-clamp, control cells were held at –80 mV (excluding Na/K pump current, see below) and membrane capacitance and membrane resistance were calculated in response to a 5 mV pulse using the Membrane Test function of Clampex. To correct for variability in cell size, current amplitudes were normalized by cell capacitance. Recordings were carried out at a sampling rate of 10 kHz, low-pass Bessel filtered at 5 kHz and stored on a computer and analyzed off-line with pClamp 10 software (Molecular Devices Inc., Sunnyvale, CA, USA).

Action potential recordings.

APs were elicited by a 1-ms current injection and measured using current clamp mode. The intracellular solution used was the same as described previously[2] (in mM): 120 K-aspartate, 25 KCl, 1 $MgCl_2$, 2 Na_2-phosphocreatine, 4 Na_2-ATP, 2 NaGTP, 10 EGTA and 5 HEPES, pH 7.2 adjusted with KOH and the bath solution was DMEM.

Sodium current recordings.

The solutions and protocol for the I_{Na} recordings were as described previously[3]. The recordings were carried out at 20 °C (TC2BIP, Cell MicroControls, Norfolk, VA, USA). The internal solution contained (in mM): 5 NaCl, 135 CsF, 10 EGTA, 5 Mg-ATP, 5 HEPES, pH 7.2 with CsOH and the bath solution contained: 20 NaCl, 117.5 CsCl, 1 $CaCl_2$, 1 $MgCl_2$, 0.1 $CdCl_2$, 11 glucose, 20 HEPES, pH 7.4 with CsOH. Series resistance was compensated down to 1 MΩ. To characterise the voltage-dependence of the peak current, cells were held at –120 mV and voltage steps were applied from –80 to +40 mV in 5 mV increments with 4-sec inter-sweep intervals. The voltage-dependence of inactivation was assessed by application of 500 ms prepulses of different voltage amplitudes (in the range from –110 mV to –40 mV), which were followed by 30 ms test pulse to –40 mV. A standard two-pulse protocol was studied to evaluate recovery rate from inactivation. Two 20 ms pulses to –40 mv separated by different time increments from 1 to 90 ms were applied.

L-type calcium current recordings.

A previously described protocol was used[4]. Shortly, L-type Ca^{2+} currents were measured using 200-ms voltage steps ranging from –30 to +50 mV in 10 mV increments at a frequency of 0.2 Hz after an initial 1-sec pre-pulse at –40 mV. The voltage dependence of inactivation was assessed by application of 2-sec pre-pulses of different voltage amplitudes (in range from –40 mV to +10 mV), which were followed by a 100-ms test pulse to +10 mV. The internal solution was (in mM): 110 CsOH, 90 aspartic acid, 20 CsCl, 10 tetraethyl ammonium chloride (TEA-Cl), 10 HEPES, 10 EGTA, 5 Mg-ATP_2, 5 Na_2-creatine phosphate, 0.4 GTP-Tris, 0.1 leupeptin, pH 7.2 adjusted with CsOH and bath solution was: 125 N-methyl-glucamine, 5 4-aminopyridine, 20 TEA-Cl, 2 $CaCl_2$, 2 $MgCl_2$, 10 glucose, 10 HEPES, pH 7.4 adjusted with HCl.

Sodium/potassium pump current recordings.

A previously described protocol was used[5]. Recording pipettes were filled with intracellular solution (in mM): 50 sodium aspartic acid, 20 potassium aspartic acid, 30 CsOH, 20 TEA-Cl, 5 $MgSO_4$, 5 HEPES, 11 EGTA, 10 glucose, 5 Na2-ATP, 1 $CaCl_2$, pH adjusted to 7.2 with CsOH and the free $[Ca^{2+}]_i$ was $1.5{\cdot}10^{-8}$ M. The ventricular myocytes were clamped at 0 mV, the saturating voltage for the Na/K pump, which could increase the signal-to-noise ratio and provide better resolution. To examine the ouabain dose-response, ouabain (10 µM and 1 mM) was applied locally, which resulted in an inward shift of the membrane current in whole cell mode.

References

1. Mutikainen, M.; Tuomainen, T.; Naumenko, N.; Huusko, J.; Smirin, B.; Laidinen, S.; Kokki, K.; Hynynen, H.; Yla-Herttuala, S.; Heinaniemi, M., *et al.* Peroxisome proliferator-activated receptor-gamma coactivator 1 alpha1 induces a cardiac excitation-contraction coupling phenotype without metabolic remodelling. *The Journal of physiology* **2016**, *594*, 7049-7071.
2. Yang, Z.; Shen, W.; Rottman, J.N.; Wikswo, J.P.; Murray, K.T. Rapid stimulation causes electrical remodeling in cultured atrial myocytes. *Journal of molecular and cellular cardiology* **2005**, *38*, 299-308.
3. Naumenko, N.; Huusko, J.; Tuomainen, T.; Koivumaki, J.T.; Merentie, M.; Gurzeler, E.; Alitalo, K.; Kivela, R.; Yla-Herttuala, S.; Tavi, P. Vascular endothelial growth factor-b induces a distinct electrophysiological phenotype in mouse heart. *Front Physiol* **2017**, *8*, 373.
4. Xu, L.; Li, X.Y.; Liu, Y.; Li, H.T.; Chen, J.; Li, X.Y.; Jiang, X.J.; Wu, G.; Tang, Y.H.; Wang, X., *et al.* The mechanisms underlying ica heterogeneity across murine left ventricle. *Molecular and cellular biochemistry* **2011**, *352*, 239-246.
5. Guo, H.C.; Guo, F.; Zhang, L.N.; Zhang, R.; Chen, Q.; Li, J.X.; Yin, J.; Wang, Y.L. Enhancement of na/k pump activity by chronic intermittent hypobaric hypoxia protected against reperfusion injury. *American journal of physiology. Heart and circulatory physiology* **2011**, *300*, H2280-2287.
